# Probiotic Mixture Protects Dextran Sulfate Sodium-Induced Colitis by Altering Tight Junction Protein Expressions and Increasing Tregs

**DOI:** 10.1155/2018/9416391

**Published:** 2018-04-15

**Authors:** Yingdi Zhang, Xiaojing Zhao, Yunjuan Zhu, Jingjing Ma, Haiqin Ma, Hongjie Zhang

**Affiliations:** Department of Gastroenterology, The First Affiliated Hospital of Nanjing Medical University, Nanjing, Jiangsu Province 210029, China

## Abstract

*Bifico* is a probiotic mixture containing *Bifidobacterium*, *Lactobacillus acidophilus*, and *Enterococcus.* Studies support that *Bifico* has a protective effect in experimental colitis (IL-10-deficient and TNBS) models and in patients with inflammatory bowel disease (IBD). However, the mechanism underlying the protective effects of this mixture of probiotic bacteria remains incompletely clear. Here, we investigated the effect of *Bifico* on intestinal inflammation. In an *in vivo* experiment, dextran sulfate sodium was used to induce colitis. *Bifico* treatment significantly attenuated the severity of colitis in this model. *Bifico* increased the expression of tight junction proteins (TJs). In addition, *Bifico* increased the number of Tregs, but reduced the number of total CD4^+^ T cells in the peripheral blood. Furthermore, the expression of colonic CD4 protein was decreased while the level of forkhead box P3 (Foxp3) was upregulated. These results suggested that *Bifico* exerts beneficial effects on experimental colitis by increasing the expressions of TJs, upregulating the number of Tregs, and reducing the total CD4^+^ T cell number in both colon and peripheral blood. The intestinal damage in the pretreated + treated-*Bifico-colitis* group was more severe than that in only the pretreated-*Bifico*-colitis group. This suggested that *Bifico* might aggravate intestinal damage when the mucosal barrier is impaired.

## 1. Introduction

The intestinal microbiota play a role in triggering the immune system and leading to intestinal inflammation [1]. IBD patients suffer from dysbiosis, which is characterized by a decrease in diversity and abundance of some dominant commensal bacteria [2]. Some studies showed that the amounts of *Lactobacillus* and *bifidobacteria* were significantly reduced in the feces of IBD patients [3, 4]. This suggests that gut flora normalization may serve as a therapeutic option for IBD patients. *Bifico* is a probiotic mixture, containing *Bifidobacterium*, *Lactobacillus*, and *Enterococcus*. In fact, evidences from both experimental studies and clinical trials have demonstrated the various therapeutic effects of *Bifico* on Crohn's disease, ulcerative colitis, colitis-induced and colitis-associated cancer in mice, pouchitis, diarrhea, and gastritis [5–12]. A previous study demonstrated that *Bifico* can increase the expression of colonic TJs and promote intestinal epithelial barrier function in interleukin-10-deficient (IL-10 KO) mice [5]. *In vitro* experiments also showed that the *Bifico*, or single probiotic strains (*Bifidobacterium*, *Lactobacillus*, or *Enterococcus*), increase transepithelial electrical resistance (TER) and the expression of TJs in enteroinvasive *Escherichia coli*- (EIEC-) treated Caco-2 monolayers. *Bifico* significantly inhibited the secretion of proinflammatory cytokines in EIEC-treated Caco-2 monolayers. *Bifico* exposure in vitro reduced bacterial invasion. Moreover, the effects of combined probiotics were more pronounced than those of single-strain probiotics. Another study reported that the anti-inflammatory effects of *Bifico* were related to the expansion of Tregs in mesenteric lymph nodes and disturbance of Th1/Th2 cytokines in the colonic mucosa of TNBS-induced colitis mice. However, the effect of *Bifico* on the Treg cells in intestinal tissue and peripheral blood has not been reported. In addition, previous animal experimental studies mainly observed the effect of *Bifico* as a therapeutic in active colitis [6].

The aims of this study were to observe the beneficial effect of *Bifico* on intestinal inflammation in dextran sodium sulfate (DSS) experimental colitis and to investigate whether the beneficial effect is associated with local and systemic immune responses. This study explored the protective effect of *Bifico* pretreatment on subsequent intestinal inflammation.

## 2. Materials and Methods

### 2.1. Animals

A total of 44 female specific-pathogen-free BALB/c mice (aged 8 to 10 weeks, weighing 20–24 g) were purchased from the Laboratory Animal Center of Nanjing Medical University (Jiangsu, China), maintained in clean cages under a 12 h light-dark cycle and conventional housing conditions, and fed with standard mouse chow. All animal experiments were performed in accordance with the National Institutes of Health Guide for the Care and Use of Laboratory Animals, and the protocol was approved by the Animal Ethics Committee of Nanjing Medical University (Approval ID: NJMU20110312). The *Bifico* capsule contains *Bifidobacterium*, *Lactobacillus*, and *Enterococcus* living bacteria not less than 3 × 10^7^ CFU (Shanghai Sine Pharmaceutical).

### 2.2. DSS-Induced Colitis and Experimental Design

**Figure 1:**
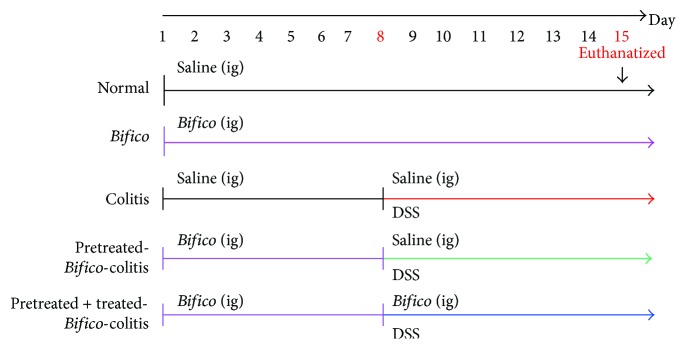
Schematic illustrations of the experimental protocols used in this study. A total of 44 female BALB/c mice were randomly assigned into 5 groups. Normal (*n* = 11), *Bifico* (*n* = 8), colitis (*n* = 8), pretreated-*Bifico*-colitis (*n* = 9), and pretreated + treated-*Bifico*-colitis (*n* = 8). ig: intragastric administration.

4% DSS (0216011080, M.W 36–50 kDa, MP Biomedicals) in drinking water was used to induce acute colitis models as reported before [13, 14]. The DSS was replaced every 2 days. Female BALB/c mice were randomly divided and treated as follows: group 1 (normal, *n* = 11): mice received sham (saline, days 1 to 14); group 2 (*Bifico*, *n* = 8): *Bifico* (days 1 to 14); group 3 (colitis, *n* = 8): DSS start on days 8–14 (saline days 1–14); group 4 (pretreated-*Bifico*-colitis, *n* = 9): *Bifico* (days 1–7) then DSS + sham (days 8–14); and group 5 (pretreated + treated-*Bifico*-colitis, *n* = 8): *Bifico* (days 1–14) and DSS added (days 8–14). At day 15, all animals were euthanatized (Figure 1). All treatments except DSS were given by oral gavage.

### 2.3. Disease Activity Index (DAI)

The DAI was determined by grading on a scale of 0–4, according to previous reports [15]: briefly, weight loss (0, ≤1%; 1, 1–5%; 2, 5–10%; 3, 10–20%; and 4, >20%), intestinal bleeding (0, negative; 2, hemoccult; 4, gross bleeding), and stool consistency (0, normal; 2, loose stools; 4, diarrhea).

### 2.4. Histological Scores

The colons were excised from the colon-cecal junction to the anus; the lengths of the colon were measured [16]. The distal colon was fixed with 10% formalin [17] and embedded in paraffin. Paraffin sections (4 *μ*m) were stained with hematoxylin-eosin (H&E). Histological scores were evaluated as previously reported [18]: inflammation (none = 0, slight = 1, moderate = 2, and severe = 3), inflamed area/extent (mucosa = 1, mucosa and submucosa = 2, and transmural = 3), crypt damage (none = 0, basal 1/3 damaged = 1, basal 2/3 damaged = 2, only the surface epithelium is intact = 3, and entire crypt and epithelium are lost = 4), and percent involvement (1–25% = 1, 26–50% = 2, 51–75% = 3, and 76–100% = 4).

### 2.5. Measurement of Tumor Necrosis Factor (TNF*α*) Levels in Colonic Tissues

Colonic tissues were homogenized in cold PBS containing a cocktail of protease inhibitors supplemented with 1 mM phenylmethylsulfonyl fluoride (PMSF). The levels of TNF*α* in colonic tissues were measured with an enzyme-linked immunoassay kit (ExCell Biology, Shanghai, China), according to the manufacturer's instructions.

### 2.6. Transmission Electron Microscopy (TEM)

Colonic tissue samples were cut into 1 mm × 1 mm × 2 mm sections and were fixed in 2.5% glutaraldehyde for 2 h at 4°C. The sections were then postfixed in 1% osmic acid for 2 h at 4°C, washed with 0.1 M PBS, dehydrated in a graded series of acetone concentrations, embedded in a mounting medium at 37°C for 3 h, and then polymerized at 60°C for 36 h. After ultrasectioning with an ultrathin slice machine (Leica, Germany), the ultrathin sections were viewed and photographed with a JEOL-1010 TEM (Japan).

### 2.7. Flow Cytometric Analysis of CD4^+^CD25^+^Foxp3^+^ T Cells

Single-cell suspensions were prepared from the peripheral blood and spleen, according to the manufacturer's protocols. Red blood cells in peripheral blood and spleen were lysed using Red Blood Cell Lysis Buffer (Beyotime, China). This step was repeated 2-3 times until no more red blood cells were visible. After that, mononuclear cells were resuspended in RPMI 1640 serum-free medium at a final cell density of 1 × 10^7^/mL. Fluorescein isothiocyanate- (FITC-) anti-mouse CD4 (RM4-5, 0.125 *μ*g/test, eBioscience) and allophycocyanin-anti-mouse CD25 (PC61.5, 0.06 *μ*g/test, eBioscience) were added to 100 *μ*L of the cell suspension for 30 min, followed by the addition of 1 mL of fixation/permeabilization working solution (eBioscience) for 10 h at 4°C. Finally, the cells were incubated with phycoerythrin- (PE-) anti-mouse/rat Foxp3 (FJK, 0.5 *μ*g/test, eBioscience) for 30 min at 4°C in the dark. Cells labeled with rat IgG2a PE were used as the isotype negative control. All flow cytometric measurements were performed on a flow cytometer (Beckman Coulter, Krefeld, Germany).

### 2.8. Immunofluorescence and Immunohistochemical Staining

Immunofluorescence staining of paraffin-embedded sections of colonic tissues was performed in accordance with routine procedures. Slides were incubated with the primary rabbit anti-Foxp3 antibody (1 : 400, Abcam, USA) overnight at 4°C, followed by FITC-labeled secondary goat anti-rabbit IgG antibody (Jackson ImmunoResearch, USA). The sections were then covered with anti-fade mounting medium (Beyotime, China) and were visualized by fluorescence microscopy. Immunohistochemical staining for TJs (JAM-1, 1 : 100, Abcam; claudin-4, 1 : 200, Abcam; occludin, 1 : 100, Proteintech) was performed as described previously [19].

### 2.9. Western Blotting

Colonic tissues were homogenized, and a total 50 *μ*g of protein was blotted onto a polyvinylidene difluoride membrane (Roche, Germany). The membranes were then incubated with specific polyclonal rabbit antibodies: anti-Foxp3 (1 : 2000, Abcam, USA), anti-IL-17 (1 : 1000, Abcam, USA), anti-JAM-1 (1 : 1000, Abcam, USA), anti-claudin-4 (1 : 300, Abcam, USA), anti-occludin (1 : 600, Proteintech, China), and monoclonal mouse anti-CD4 (1 : 100, Abcam, USA). The secondary antibodies were incubated at room temperature for 1 h. Data were analyzed by ImageLab2.0.1 software and normalized to GAPDH expression.

### 2.10. RNA Isolation and Quantitative RT-PCR

Total RNA from the colonic tissues was extracted using TRIzol® reagent (Life Technologies). The total RNA concentration was measured with a BioPhotometer (Eppendorf, Hamburg, Germany). A total of 1 *μ*g total RNA was reverse-transcribed into cDNA using oligi (dT) 18 primers (TaKaRa). The primers were as follows: mouse IL-17 sense, 5′-CTCCAGAAGGCCCTCAGACTAC-3′; antisense, 5′-AGCTTTCCCTCCGCATTGACACAG-3′; and mouse *β*-actin sense, 5′-ACCACCATGTACCCAGGCATT-3′; antisense, 5′-CCACACAGAGTACTTGCGCTCA-3′. Following the reactions, cycle threshold values were determined by setting a fixed threshold. The relative amount of IL-17 mRNA was normalized to the reference gene *β*-actin.

### 2.11. Statistical Analysis

All images are representative of at least three independent experiments. Data are presented as the mean ± standard error of the mean (SEM). *P* values were calculated by one-way ANOVA followed by the Tukey post hoc test (SPSS version 21.0). *P* value < 0.05 was considered significant.

## 3. Results

### 3.1. Bifico Attenuated Colitis in Mice

**Figure 2:**
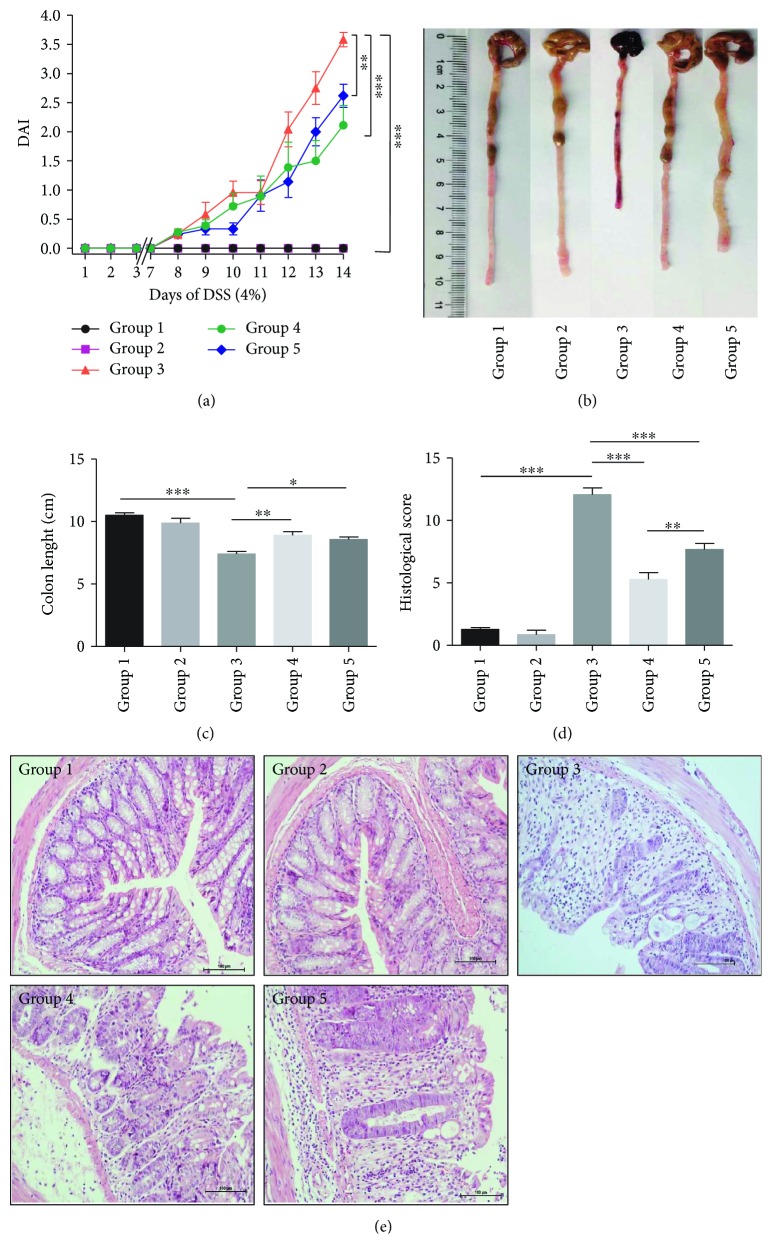
Evaluation of treatment efficacy in colitis mice. (a) DAI was determined in each group of mice. (*n* = 8–11/group). (b) and (c) Macroscopic observation of the colon. Colon length measured in cm. (d) Histological scores were evaluated in colons. (e) Typical histological images (200x magnification). Data are means ± SEM. ^∗^*P* < 0.05, ^∗∗^*P* < 0.01, and ^∗∗∗^*P* < 0.001. Group 1: normal; group 2: *Bifico*; group 3: colitis; group 4: pretreated-*Bifico*-colitis; group 5: pretreated + treated-*Bifico*-colitis.

The DAI scores in pretreated-*Bifico*-colitis and pretreated + treated-*Bifico*-colitis groups dramatically decreased compared with the colitis group (*P*_1_ < 0.001 and *P*_2_ < 0.01) (Figure 2(a)). The mean lengths of the colon were significantly improved in pretreated-*Bifico*-colitis and pretreated + treated-*Bifico*-colitis groups compared with the colitis group (*P*_1_ < 0.01 and *P*_2_ < 0.05, resp.) (Figures 2(b) and 2(c)). Furthermore, the villus necrosis, hemorrhage, and inflammatory cell infiltrates in the lamina propria were shown in colonic tissues of colitis mice. *Bifico* treatment drastically alleviated inflammatory cell infiltrates in the colon. Meanwhile, the values of colonic histological score in pretreated-*Bifico*-colitis and pretreated + treated-*Bifico*-colitis groups drastically decreased compared with the colitis group (*P* < 0.001 for both) (Figures 2(d) and 2(e)).

### 3.2. Administration of Bifico Reduced Colonic Levels of TNF*α* in Colitis Mice

**Figure 3:**
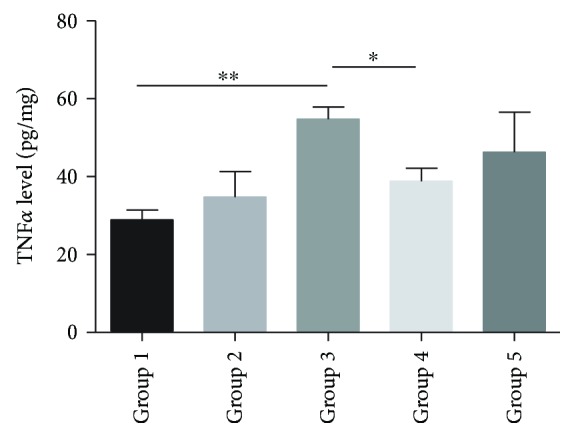
Administration of *Bifico* and concentration of TNF*α*. The colonic levels of TNF*α* were increased in the colitis group (3) compared to the normal group (1) (*P* < 0.01) and decreased in the Pretreated-*Bifico*-colitis group (4) and pretreated + treated-*Bifico*-colitis group (5) (*P*_1_ < 0.05 and *P*_2_ = 1.00, resp.). Data are means ± SEM (*n* = 8–11/group). ^∗^*P* < 0.05 and ^∗∗^*P* < 0.01.

As reported previously, the colonic levels of TNF*α* were increased in the colitis group (*P* < 0.01). Meanwhile, the level of TNF*α* in the pretreated-*Bifico*-colitis group was decreased compared to the colitis group (*P* < 0.05). However, there was no significant difference between the colitis group and the pretreated + treated-*Bifico*-colitis group. Notably, the colonic levels of TNF*α* were slightly increased in the *Bifico* group, but there was no significance compared with the normal group (*P* = 0.934) (Figure 3).

### 3.3. Alterations in Microstructures of the Colonic Epithelial Barrier in Colitis Mice Observed by TEM

**Figure 4:**
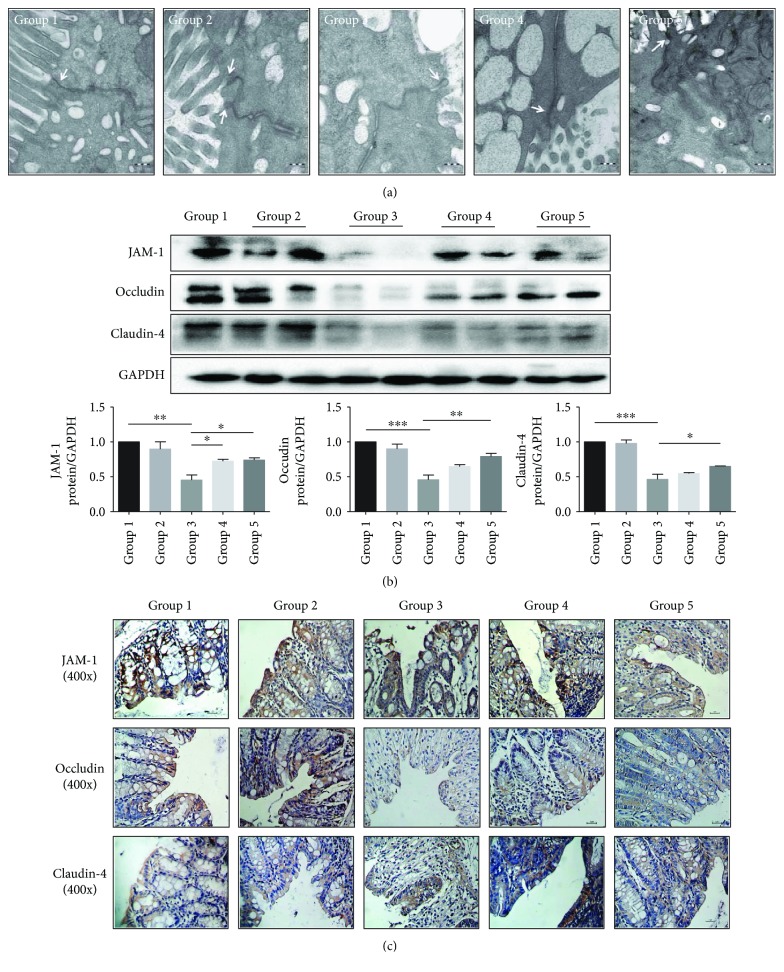
The effect of *Bifico* on the expression and distribution of TJs (a). Microstructural changes in colonic tissues of each group by TEM (80000x magnification) (b) The expressions of JAM-1, occludin, and claudin-4 were detected by Western blot. Group 1 (*n* = 3), group 2 (*n* = 6), group 3 (*n* = 6), group 4 (*n* = 6), and group 5 (*n* = 6). (c) The expressions and distribution of TJs (JAM-1, occludin, and claudin-4) were analyzed by immunohistochemistry (400x magnification). Data are means ± SEM. ^∗^*P* < 0.05, ^∗∗^*P* < 0.01, and ^∗∗∗^*P* < 0.001. Group 1: normal; group 2: *Bifico*; group 3: colitis; group 4: pretreated-*Bifico*-colitis; group 5: pretreated + treated-*Bifico*-colitis.

The morphology of the colonic epithelium and the cell membrane was intact, the cell junction was tight, and the villi on the cell surfaces were well-arranged in both the normal group and *Bifico* groups. However, in the colitis group, the integrity of the epithelial membranes was compromised, cell-cell junctions were loose, with an obvious intercellular space broadening, and villi on the cell surfaces were decreased (Figure 4(a)). The microstructure of the cell-cell junction was improved in pretreated-*Bifico*-colitis and pretreated + treated-*Bifico*-colitis groups, and some villi on the surfaces were reserved.

### 3.4. Expressions and Distribution of TJs in Colonic Tissues and Epithelium Barrier

Compared with the normal group, the expressions of the TJs were significantly reduced in the colitis group (*P* < 0.01 for JAM-1, *P* < 0.001 for occludin, and *P* < 0.001 for claudin-4). The expression levels of these TJs in pretreated-*Bifico*-colitis and pretreated + treated-*Bifico*-colitis groups were extensively increased compared with the colitis group (*P*_1_ < 0.05 and *P*_2_ < 0.05 for JAM-1, *P*_1_ = 0.073 and *P*_2_ < 0.01 for occludin, and *P*_1_ = 0.598 and *P*_2_ < 0.05 for claudin-4). In addition, the expression levels of occludin and claudin-4 in the pretreated + treated-*Bifico*-colitis group were slightly higher than those in the pretreated-*Bifico*-colitis group with no significant difference (*P* = 0.246 for occludin and *P* = 0.345 for claudin-4) (Figure 4(b)). There was no difference between the *Bifico* group and the normal group, but a scattered distribution and decreased expressions of TJs were found in colitis mice. However, the intensity and the percentage of cells stained for TJs were improved in pretreated-*Bifico*-colitis and pretreated + treated-*Bifico*-colitis groups, in comparison with mice in the colitis group (Figure 4(c)).

### 3.5. Bifico Effects on Total CD4^+^ T Cells, CD4 Protein Expression, Tregs, and Foxp3^+^ Cells

**Figure 5:**
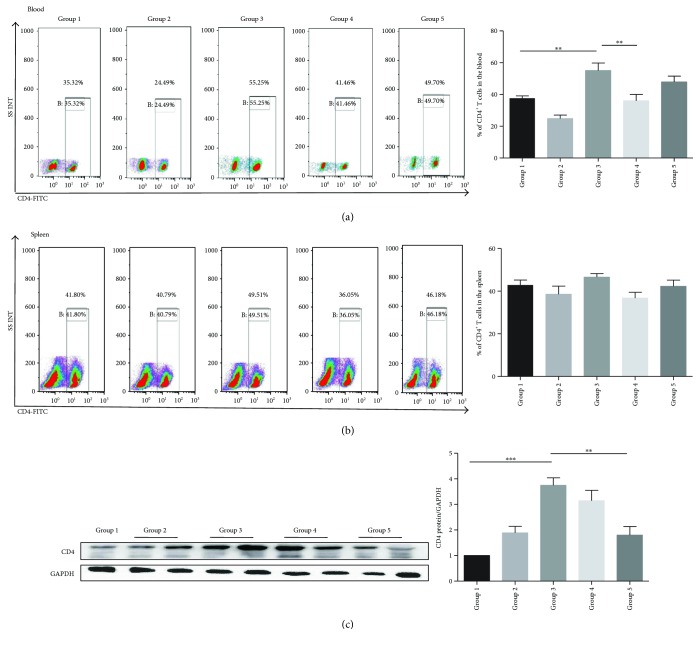
The proportion of total CD4^+^ T cells in the peripheral blood and spleens in each group. (a) The frequency of CD4^+^ T cells in the blood. (b) The proportion of total CD4^+^ T cells in the spleen (*n* = 6–9/group). The number of Foxp3^+^ cells in colonic tissues (the white arrows indicate Fox3^+^ cells) (*n* = 6–9/group). (c) Western blot analysis of CD4 protein expression levels. Group 1 normal (*n* = 3); group 2 *Bifico* (*n* = 6); group 3 colitis (*n* = 6); group 4 pretreated-*Bifico*-colitis (*n* = 6); group 5 pretreated + treated-*Bifico*-colitis (*n* = 6). Data are means ± SEM. ^∗∗^*P* < 0.01 and ^∗∗∗^*P* < 0.001.

**Figure 6:**
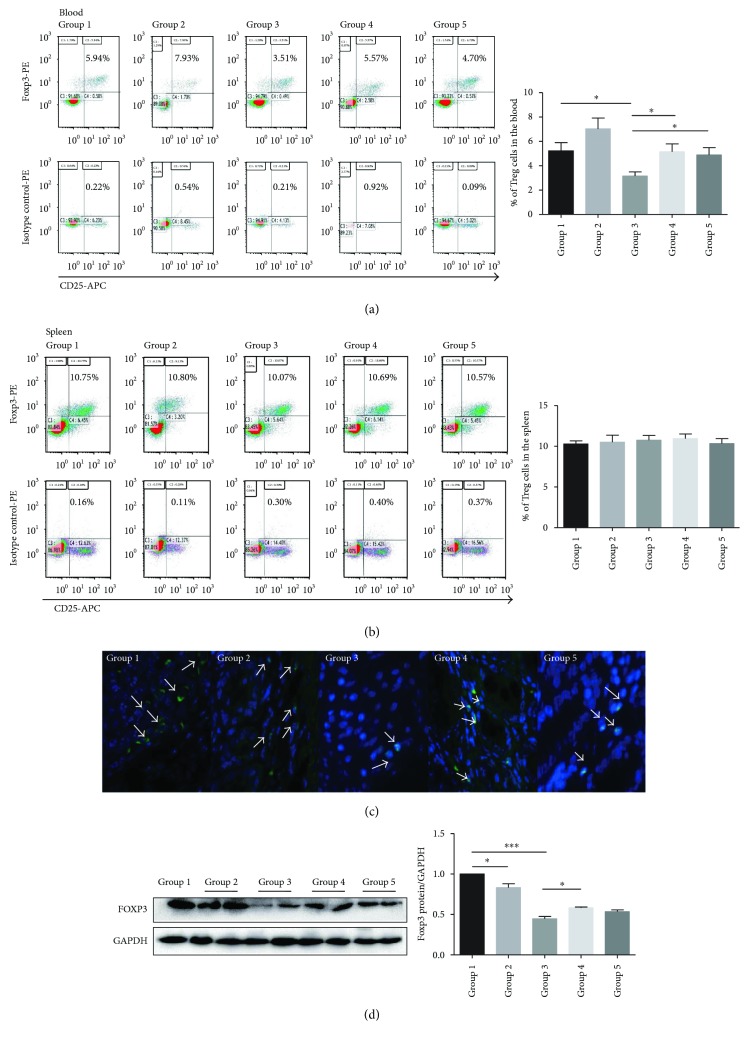
The proportion of total CD4^+^CD25^+^Foxp3^+^ Tregs cells in the peripheral blood and spleen. (a) The frequency of Treg cells in the blood (*n* = 6–9/group) (b) and in the spleen (*n* = 6–9/group). (c) The number of Foxp3^+^ cells in colonic tissue (the white arrows indicate Fox3^+^ cells). (d) Western blot analysis of mice colonic Foxp3 protein expression level in each group. Group 1 normal (*n* = 3); group 2 *Bifico* (*n* = 6); group 3 colitis (*n* = 6); group 4 pretreated-*Bifico*-colitis (*n* = 6); group 5 pretreated + treated-*Bifico*-colitis (*n* = 6). Data represent means ± SEM. ^∗^*P* < 0.05 and ^∗∗∗^*P* < 0.001.

The proportion of total CD4^+^ T cells in the peripheral blood in the colitis group was increased (colitis versus normal group: 55.10 ± 4.73 versus 34.19 ± 1.49, *P* < 0.01); however, compared with the colitis group, the increased proportion of total CD4^+^ T cells was partially reversed both in pretreated-*Bifico*-colitis (36.04 ± 3.99 versus 55.10 ± 4.73, *P* < 0.01) and pretreated + treated-*Bifico*-colitis groups (47.90 ± 3.66 versus 55.10 ± 4.73, *P* = 0.556) (Figure 5(a)). There was no significant difference in the number of splenic CD4^+^ T cells among different groups (Figure 5(b)). In addition, there was a decreasing trend of the colonic CD4 protein level in the pretreated-*Bifico*-colitis group compared with the colitis group (*P* = 0.639). Meanwhile, a significant difference was shown between pretreated + treated-*Bifico*-colitis and colitis groups (*P* < 0.01) (Figure 5(c)). In addition, the population of CD4^+^CD25^+^Foxp3^+^ Tregs in the peripheral blood was significantly upregulated in the pretreated-*Bifico*-colitis and pretreated + treated-*Bifico*-colitis groups compared with the colitis group (*P* < 0.05 for both) (Figure 6(a)). However, no significant difference in the amount of CD4^+^CD25^+^Foxp3^+^ Tregs in the spleen was detected among groups (Figure 6(b)). In fact, the number of Foxp3^+^ Tregs in colonic tissue was increased by the administration of *Bifico* in pretreated-*Bifico*-colitis and pretreated + treated-*Bifico*-colitis groups compared with the colitis group (Figure 6(c)). The expression of Foxp3 protein was significantly decreased in the colitis group compared with the normal group (*P* < 0.001) as well as the pretreated-*Bifico*-colitis group (*P* < 0.05). Notably, the expressions of colonic Foxp3 protein were slightly increased in the pretreated + treated-*Bifico*-colitis group, but was not significant (*P* = 0.224) (Figure 3 versus (Figure 6(d)). However, compared with the normal group, the expression of Foxp3 protein in the *Bifico* group was decreased (*P* < 0.05).

### 3.6. Bifico Modulated the Expression of IL-17

**Figure 7:**
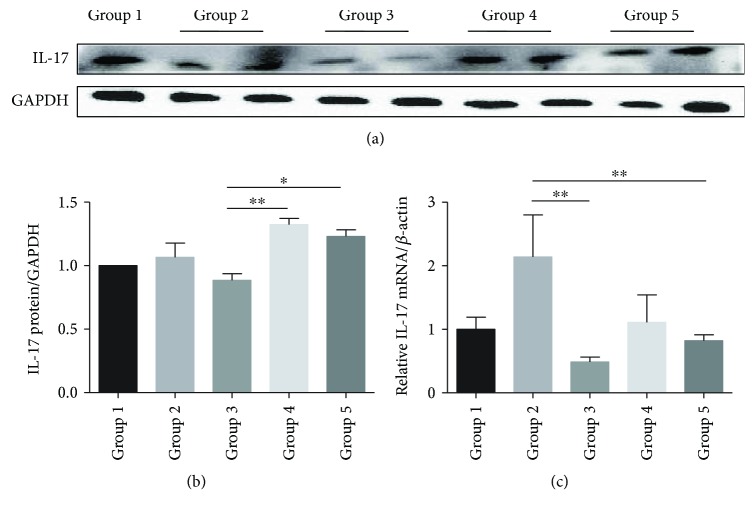
*Bifico* modulated the expression of IL-17. (a, b) Western blot analysis of IL-17 protein expression levels in colonic tissues. Group 1 normal (*n* = 3); group 2 *Bifico* (*n* = 6); group 3 colitis (*n* = 6); group 4 pretreated-*Bifico*-colitis (*n* = 6); group 5 pretreated + treated-*Bifico*-colitis (*n* = 6). (c) The levels of IL-17 mRNA (*n* = 8–11/group). Data are means ± SEM_._^∗^*P* < 0.05 and ^∗∗^*P* < 0.01.

There was an increasing trend for the colonic IL-17 mRNA expression in the pretreated-*Bifico*-colitis and pretreated + treated-*Bifico*-colitis groups (*P*_1_ < 0.01 and *P*_2_ < 0.05, resp.) and a decrease in the colitis group (Figures 7–7) (*P* = 0.780).

## 4. Discussion

Some studies suggested that *Bifico* supplements are able to prevent colitis development in human IBD and experimental colitis [5–7, 20]. The current study shows that the intestinal damage in the pretreated + treated-*Bifico*-colitis group was more severe compared with that in the pretreated-*Bifico*-colitis group, suggesting that *Bifico* might have a protective effect on colonic tissue in the intact intestinal mucosal barrier. In contrast, when the mucosal barrier is impaired, probiotics might aggravate colonic tissue damage, and this needs further research. The TJs consist of transmembrane proteins such as occludin, claudins, junctional adhesion molecules, and adaptor proteins like Zos [21]. An oral administration of *Bifico* has been shown to reduce colon inflammation and to protect epithelial barrier function in IL-10 KO mice [5]. Consistent with these findings, this study shows that *Bifico* increased the expressions of TJs and improved the microstructures in colitis mice. Previous investigators showed that *Bifico* treatment significantly reduced the levels of TNF*α* in the colon of experimental colitis mice [5, 6]. Our data support these findings that *Bifico* may ameliorate intestinal inflammation by inhibiting TNF*α* production.

Foxp3 is required for induction of the immune suppressive function of CD4^+^Foxp3^+^Tregs and maintenance of mucosal immune homeostasis by regulating the balance between CD4^+^Foxp3^+^Tregs and helper effector T cells. Tregs are critically involved in the prevention of human IBD and experimental colitis [6, 22]. A previous study has shown that the anti-inflammatory effects of *Bifico* were related to the expansion of Tregs in mesenteric lymph nodes of colitis mice and the ratio of Th1/Th2 might be regulated by Tregs [6]. We found that *Bifico* increased the number of Tregs in the peripheral blood. However, the numbers of Tregs in the spleen among groups showed no significant differences. Meanwhile, *Bifico* could increase the colonic Foxp3 protein level, but decrease the colonic CD4 protein level in colitis mice. These results may indicate that the decreased frequency of Treg subsets in the peripheral blood and Foxp3 expression in the colonic tissues might destroy the intestinal immune tolerance and activate inflammation. However, a significant investigation is required to prove these explanations.

Of note, the normal mice treated with *Bifico* demonstrated a slightly increased TNF*α* level, a decreased Foxp3 protein level, and an increased CD4 protein expression in colonic tissues as well as an increased number of Tregs in the peripheral blood. It is possible that *Bifico* acts as a foreign antigen and triggers weak immune responses, yet this immune response may not lead to pathological inflammatory damage. This suggested that proper immune activation may enhance the mucosal defense and thus be beneficial for the promotion of the host intestinal immunity [23]. These also need to be investigated.

Some studies have demonstrated that IL-17 is an important proinflammatory cytokine and is highly expressed in the inflamed gut in IBD patients and colitis mice [24, 25]. In contrast, other studies have suggested that IL-17A has a protective role in a T-cell transfer model of colitis [26, 27]. Furthermore, the severity of murine colitis was enhanced with anti-IL-17 neutralizing antibody treatment or IL-17A knockout [28, 29]. In our study, the expressions of IL-17 mRNA and protein were decreased in colitis mice, but were increased after *Bifico* intervention. This is in agreement with a previous study, which showed that the expression of IL-17 in colitis mice was increased treated with *Bifidobacterium breve* [16]. The production of splenic IL-17 is reduced by DSS [30]; however, the precise roles of IL-17 in the development of IBD need to be elucidated in future studies.

Our study has some strengths, as *Bifico* might have a protective effect on colonic tissue when the intestinal mucosal barrier is intact. In contrast, when the mucosal barrier is impaired, probiotics might aggravate colonic tissue damage. *Bifico* could increase the number of Tregs in the peripheral blood and have no influence in the spleen among different groups. Our study has several limitations. First, TJ expression is only an indirect reflection of the barrier function. To exactly measure the barrier function, physiologic measurements like absorption of orally administered dextran or bacteria-size particles should be performed. Second, this study explored the effects of *Bifico* as a whole, but functions of single probiotic strains contained in *Bifico* (*Bifidobacterium*, *Lactobacillus*, *or Enterococcus*) were not studied separately. Third, these results suggested that *Bifico* might exert beneficial effects on experimental colitis by upregulating the number of Tregs and reducing total CD4^+^ T cells in both colonic tissue and peripheral blood. Further, pathways through which Tregs and CD4^+^ T cells ameliorate the inflammation need to be explored. Finally, this study only explored the preventive effects of *Bifico* (with no major clinical application) while no study was performed to explore therapeutic effects in this model. Therefore, extensive exploration in this field is needed.

## 5. Conclusions

This study demonstrated some beneficial effect of *Bifico* on colitis. The potential mechanism involved in improving the expression of TJs is increasing the number of Tregs in colonic tissues and the peripheral blood, while decreasing the proportions of total CD4^+^ T cells in colonic tissues and the peripheral blood.

## Abbreviations

IBD: Inflammatory bowel disease
DSS: Dextran sulfate sodium
TJs: Tight junction proteins
JAM-1: Junctional adhesion molecule 1
Foxp3: Forkhead box P3
IL-17: Interleukin-17
IL-10: Interleukin-10
DAI: Disease activity index
H&E: Hematoxylin-eosin
TNF*α*: Tumor necrosis factor
PMSF: Phenylmethylsulfonyl fluoride
TEM: Transmission electron microscopy
FITC: Fluorescein isothiocyanate
PE: Phycoerythrin
SEM: Standard error of the mean.
